# Modelling *gambiense* human African trypanosomiasis infection in villages of the Democratic Republic of Congo using Kolmogorov forward equations

**DOI:** 10.1098/rsif.2021.0419

**Published:** 2021-10-06

**Authors:** Christopher N. Davis, Matt J. Keeling, Kat S. Rock

**Affiliations:** ^1^ Mathematics Institute, University of Warwick, Coventry CV4 7AL, UK; ^2^ Zeeman Institute (SBIDER), University of Warwick, Coventry CV4 7AL, UK; ^3^ School of Life Sciences, University of Warwick, Coventry CV4 7AL, UK

**Keywords:** mathematical model, Kolmogorov forward equations, African trypanosomiasis, African sleeping sickness

## Abstract

Stochastic methods for modelling disease dynamics enable the direct computation of the probability of elimination of transmission. For the low-prevalence disease of human African trypanosomiasis (gHAT), we develop a new mechanistic model for gHAT infection that determines the full probability distribution of the gHAT infection using Kolmogorov forward equations. The methodology allows the analytical investigation of the probabilities of gHAT elimination in the spatially connected villages of different prevalence health zones of the Democratic Republic of Congo, and captures the uncertainty using exact methods. Our method provides a more realistic approach to scaling the probability of elimination of infection between single villages and much larger regions, and provides results comparable to established models without the requirement of detailed infection structure. The novel flexibility allows the interventions in the model to be implemented specific to each village, and this introduces the framework to consider the possible future strategies of test-and-treat or direct treatment of individuals living in villages where cases have been found, using a new drug.

## 1. Background

In mathematical epidemiology, a growing number of models employ the use of stochastic events to describe infection dynamics. These stochastic methodologies, such as the Gillespie or tau-leaping algorithm, typically use a large number of event-driven stochastic simulations to estimate a distribution of possible behaviours for an epidemic [1]. A central benefit of stochastic models is that the random nature of each realization means a larger range of outcomes is captured than simply the equilibrium dynamics predicted by a deterministic model [2]. These stochastic methods are also integer-based and so the exact number of people infected at a given time is monitored, including when infections reach zero; a deterministic model will never reach zero infections and so a threshold needs to be applied to reach the elimination [3–5]. Thus, a model in a stochastic framework, will have different expected behaviour and be better suited than a deterministic variant when close the the elimination threshold, either due to low infection numbers or small populations [6].

However, event-driven stochastic methods require a large number of realizations of the epidemic process to be generated to be confident that the full distribution of events has been captured; even then this is still only an approximation of the true probability distribution of the potential trajectories for the infection dynamics in time. This is particularly important if there are any rare events of the system—something that requires more realizations to determine the true frequency at which they occur [6]. Alternative to these event-driven stochastic simulations, the Kolmogorov forward equations provide a method incorporating the stochastic behaviour in a set of ordinary differential equations (ODEs), which fully determine the probability distribution of the epidemic over all possible infection states for the population [6]. This approach is simple to formulate, as many systems are linear in terms of the probability of being in each infection state, and so can be written in a matrix formulation; other methods exist for when this is not the case [7]. The system is thus easy to solve using standard methods and provides a complete description of the dynamics. It is also much faster to solve than repeatedly generating realizations of the stochastic process. The solutions are also numerically exact and an array of derived quantities can be directly calculated.

Kolmogorov forward equations have been used in several epidemiological contexts [8–10] and discussed as a powerful tool, but are not widely used due to constraints on computer memory [11]. Every possible infection state must be explicitly tracked and so if a population is large (and particularly if each individual can be in any of a large number of infection states), the number of these states quickly becomes prohibitive to the method, as the number of required computations becomes infeasible. Simulation methods take a lot of computation time; Kolmogorov forward equation methods take a lot of memory storage capacity [12]. Therefore, a Kolmogorov forward equation approach to modelling infections dynamics would be most applicable for a disease with a relatively small number of cases that has the potential to achieve elimination [13]. One such disease is *gambiense* human African trypanosomiasis (gHAT).

Infection with gHAT is caused by the parasite *Trypanosoma brucei gambiense* and is transmitted by tsetse across Central and West Africa, with the majority of infection occurring in the Democratic Republic of Congo (DRC). This infection has been traditionally controlled with active and passive screening, with subsequent treatment of infected individuals, and has been targeted for elimination of transmission by 2030 by the World Health Organization (WHO). There have been a substantial number of recent modelling studies that use compartmental models for gHAT, with these studies typically using deterministic systems of ODEs [14–18], with more recent studies also considering stochastic event-driven approaches [19–22].

Here, we have developed new model for gHAT infection in spatial connectivity of villages within health zones of DRC. We use a lower-dimensional state space for infection than considered in the majority of the literature, which hence allows for Kolmogorov forward equations to be used to define the dynamics. This formulation fully captures the possible behaviour of the infection in a stochastic framework, while exhibiting the advantages of providing the full and exact distribution of the infection states. We investigate the probabilities of gHAT persistence or extinction and compute expected times until elimination of infection, which, despite the lower-dimensional infection structure, provides comparable results to more commonly used methods.

Furthermore, the Kolmogorov forward equation model provides the necessary formulation to explore the interactions between a large number of villages. Village-specific simulations can mimic the real-world interventions observed at the level at which they occur, with the results then realistically scaled up to larger regions, where elimination of infection can be considered at more meaningful geographical scales [23]. In the context of gHAT, the method provides a link between individual village [19] and health zone [18] modelling, while including the stochastic properties required to directly simulate elimination of infection, we can assess potential strategies and progress towards achieving elimination goals.

## 2. Methods

### 2.1. Kolmogorov forward equations

We construct our Kolmogorov forward equation model by adapting the structure of the suite of gHAT models first presented in Rock *et al.* [14] and updated in Crump *et al.* [17]. Unlike these previous models, the model presented in this paper contains just two infection states for a person (susceptible and infected) and two types of people (low- and high-risk of exposure to tsetse bites), and does not explicitly model the number of infected tsetse, the biological vector of the disease.

**Figure 1.**
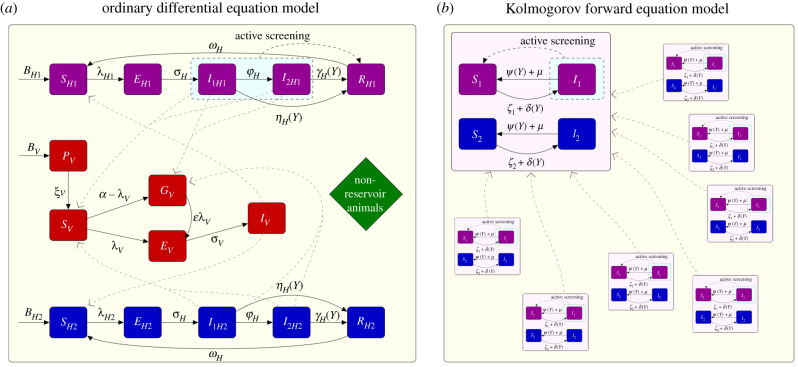
Model diagrams for the two gHAT models. (*a*) The full higher-dimensional ODE model from Crump *et al.* [17] that includes tsetse dynamics. (*b*) The lower-dimensional spatially connected Kolmogorov forward equation model presented in this paper. In (*b*), the larger box represents an example village with the smaller boxes being the other villages (which have identical structure). In (*a*,*b*), the solid black lines show the rate of movement between compartments and dashed black lines show the the result of active screening and treatment of infected individuals. The dashed grey paths show interaction between humans and tsetse or between all villages, respectively in each panel.

We derive the new model equations by replacing the tsetse dynamics of previous models with the quasi-equilibrium solution, in order to limit the number of model compartments. For the case gHAT, this assumption is justified by the short life-expectancy of the vector (tsetse) [24] and the long timescales of the infection in humans [25]. The number of infection compartments for humans are also reduced from five (susceptible, exposed, infected Stage 1, infected Stage 2 and hospitalized) to two, whereby exposed, infected Stage 1 and infected Stage 2 are now included as a single infected state, *I*, and the former susceptible and hospitalized compartments are now given as all susceptible *S* (figure 1). The total human population size is constant and denoted as *N*, with a small natural mortality rate of people, *μ*, replaced by new susceptible individuals. We retain a risk structure whereby a small minority of the population is high-risk, with a higher exposure to biting tsetse and failure to attend active screening.

This leaves four possible infection states for any person, and therefore this lower-dimensional model structure (shown in an ODE framework) is given by equations (2.1) and (2.2):

2.1 $$\frac{dS_{i}}{dt}=(\mu +\psi (Y))I_{i}-\zeta_{i}(I_{1},I_{2})S_{i}$$

and

2.2 $$\frac{dI_{i}}{dt}=\zeta_{i}(I_{1},I_{2})S_{i}-(\mu +\psi (Y))I_{i},$$

for *i* = 1, 2, for the low- and high-risk group, respectively. The natural human mortality rate, *μ*, determines the total human birth rate, *μN*, such that a constant population size is maintained. The recovery rate, *ψ*(*Y*), is dependent on time due to increased detection rates at later times and is derived from a combination of parameters from Crump *et al.* [17], detailed in the electronic supplementary material. The force of infection is given by *ζ*_*i*_(*I*_1_, *I*_2_), which is a function of the risk class and the number of people infected in each risk class, further depending on the quasi-equilibrium solution for the tsetse (figure 1). See electronic supplementary material a full explanation of the derivation for these model parameters.

To translate this re-formulated model (equations (2.1) and (2.2)) into the Kolmogorov forward equations, we first consider infection state of the population. Since we assume constant population sizes *N*_1_ and *N*_2_ in each risk group and *S*_*i*_ + *I*_*i*_ = *N*_*i*_ for *i* = 1, 2, the infection state is fully determined by the number of infected low- and high-risk people *I*_1_ and *I*_2_. Thus, we define the probability of being in a given state at time *t* as $P_{I_{1},I_{2}}(t)$. From this population infection state, there are four possible transitions to different states: a low-risk human can recover or die, a high-risk human can recover or die, a low-risk human can get infected, or a high-risk human can get infected (since we assume a constant population size, death is followed by immediate replacement in the susceptible class and so we do not consider this separately from recovery). Therefore, the Kolmogorov forward equations are given by

2.3 $$\begin{array}{rl} \frac{dP_{I_{1},I_{2}}(t)}{dt} & =-P_{I_{1},I_{2}}(t)(\zeta_{1}(I_{1},I_{2},t)(N_{1}-I_{1})\\ & \;+\zeta_{2}(I_{1},I_{2},t)(N_{2}-I_{2})+(\mu +\psi (Y))(I_{1}+I_{2}))\\ & \;+P_{I_{1}-1,I_{2}}(t)\zeta_{1}(I_{1}-1,I_{2},t)(N_{1}-I_{1}+1)\\ & \;+P_{I_{1},I_{2}-1}(t)\zeta_{2}(I_{1},I_{2}-1,t)(N_{2}-I_{2}+1)\\ & \;+P_{I_{1}+1,I_{2}}(t)(\mu +\psi (Y))(I_{1}+1)\\ & \;+P_{I_{1},I_{2}+1}(t)(\mu +\psi (Y))(I_{2}+1), \end{array}$$

where *I*_1_ = 0, …, *M*_1_, *I*_2_ = 0, …, *M*_2_, and $P_{I_{1},I_{2}}(t)=0$ for *I*_1_ < 0 and *I*_2_ < 0. The values *M*_1_ and *M*_2_ are the maximum number of people possible to be infected in each risk group.

In theory, *M*_1_ = *N*_1_ and *M*_2_ = *N*_2_; however because in practice gHAT is a low-prevalence infection [26], we can reduce the state space of the model and impose a lower maximum threshold, while retaining high model accuracy. We ensure that the probability of exceeding the threshold is very small (less than 1 × 10^−8^), with the values of *M*_*i*_ dependent on *N*_*i*_, and explicitly given in the electronic supplementary material.

Thus, the Kolmogorov forward equations (equation (2.3)) comprise of a system of (*M*_1_ + 1)(*M*_2_ + 1) ODEs (since the low-risk infected population can take any value between 0 and *N*_1_ and similarly for the high-risk). The Kolmogorov forward equations are a linear system, and hence we simplify the notation by writing the equations in matrix form. By defining the probability vector,

2.4 $$p(t)=(P_{0,0}(t),P_{1,0}(t),\ldots ,P_{N_{1},0}(t),P_{0,1}(t),\ldots ,P_{N_{1},N_{2}}(t)),$$

we obtain the Kolmogorov forward equations in matrix form as

2.5 $$\frac{dp(t)}{dt}=p(t)Q(t),$$

where **Q**(*t*) is the rate matrix of all transition rates at time *t*. We subsequently give the time *t* in both the year *Y* and number of days into that year *d* and hence, **p**(*t*) = **p**(*Y*, *d*). However, we note that **Q**(*t*) = **Q**(*Y*) because we assume, as per Crump *et al.* [17], that the change in the rate matrix is due to an increase in the rate of passive detection of infection due to improvements in the passive surveillance system, which occurs annually.

Since the equations of Crump *et al.* [17] consider large populations of roughly 100 000 people, rather than much smaller villages, we additionally include a rate of importation of infection into a village, due to movement of people between villages, for which we use a value derived in Davis *et al.* [19] and denote by *δ*(*Y*). The event of an external importation increases the number of infected people in either risk group by one and adds terms to equation (2.3) representing a change of state from *I*_1_ low-risk infected and *I*_2_ high-risk by increasing from *I*_1_ − 1 to *I*_1_, increasing from *I*_2_ − 1 to *I*_2_, and increasing from *I*_1_ and *I*_2_ to *I*_1_ + 1 or *I*_2_ + 1 respectively:

2.6 $$\begin{array}{rl} & +P_{I_{1}-1,I_{2}}(t)\delta (Y)(N_{1}-I_{1}+1)\\ & +P_{I_{1},I_{2}-1}(t)\delta (Y)(N_{2}-I_{2}+1)\\ \mathrm{and}\;\; & -P_{I_{1},I_{2}}(t)\delta (Y)((N_{1}-I_{1})+(N_{2}-I_{2})). \end{array}\}$$

In matrix notation, we include these new terms in matrix **Q**_*E*_(*Y*), whereby the rate matrix is given by **Q**(*Y*) = (**Q**_*V*_(*Y*) + **Q**_*E*_(*Y*)), for the village and external terms respectively.

Additionally, active screening, the process whereby a large number people in a village are targeted to be screened for the disease and then treated if infected, is modelled as a multiplication of the probability vector **p**(*Y, y*) by a lower-triangular transition matrix **A**(*Y*). In line with previous modelling studies, we assume that only the low-risk class are affected by this discrete-time event that occurs annually at the beginning of each year, whereas the high-risk class do not attend active screening events. The active screening matrix **A**(*Y*) is calculated, for a given screening coverage (which can change each year), by the use of a hypergeometric distribution to determine the number of infected people screened, followed by a binomial distribution to find the number of infections detected due to the imperfect sensitivity of the test (full details are in the electronic supplementary material).

We assume that active screening began in 1998 [27], and the system was previously at endemic equilibrium given there had been no screening for several years previously. Therefore, we derive the full distribution of infection states for a population in day *d* of year *Y* as

2.7 $$\begin{array}{rl} p(Y,d) & =p(1997,365)\left(\prod_{i=1998}^{Y-1}A(i)\exp(-365(Q_{V}(i)+Q_{E}(i)))\right)\\ & \;\times A(Y)\exp(-d(Q_{V}(Y)+Q_{E}(Y))), \end{array}$$

for *Y* ≥ 1998 and 0 ≤ *d* ≤ 365. Active screening in year *Y* is modelled as occurring at the start of the year (*d* = 0), and hence the probability state just before active screening in year *Y* is given by **p**(*Y* − 1, 365). We calculate **p**(1997, 365) by finding quasi-stationary equilibrium, finding the eigenvector corresponding to the largest eigenvalue of the rate matrix, (**Q**_*V*_(1997) + **Q**_*E*_(1997)).

### 2.2. Parameter values

The parameters are taken from Crump *et al.* [17] and transformed into the new parameters of the Kolmogorov forward equations (see electronic supplementary material). The values of the original parameters are either fixed where well-defined in the literature, and specific to DRC, or are parameters reflective of a high-incidence health zone (by using the median estimate of the posterior from fitting to screening and incidence data from the health zone Kwamouth in Mai Ndombe province using a Metropolis–Hastings Markov chain Monte Carlo algorithm [17]). The data which span 2000–2016 came from the WHO HAT Atlas [26].

In the present study, we do not account for vector control, as our primary focus here is to understand the village-level screening dynamics. Furthermore, only one health zone of DRC had large-scale vector control *in situ* prior to 2018 [28] (the study period with available data).

We additionally present similar results for the low-incidence exemplar health zone (based on parameterization from Mosango health zone of Kwilu province) for comparison in figure 5 and in the electronic supplementary material. We note that the methodology presented here could alternatively be applied to any other health zone or region.

## 3. Results

### 3.1. Infection dynamics of a single village

**Figure 2.**
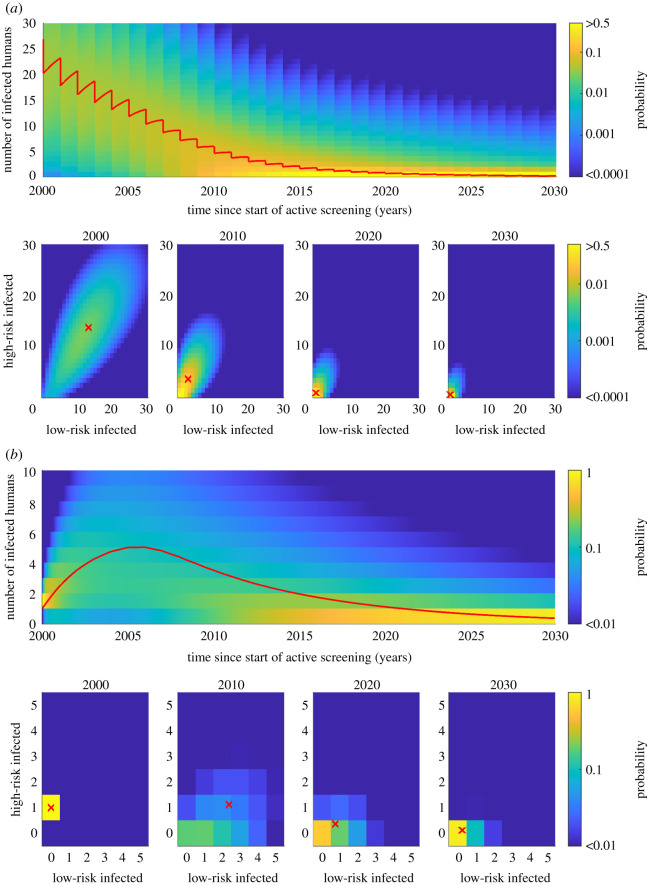
Illustrative example of the distribution of infection in a single example village of population size *N* = 1000 for two scenarios. (*a*) A gHAT-endemic village with 50% annual active screening coverage and a small rate of infectious importations. (*b*) A village with local elimination of infection pre-2000 followed by the introduction of one high-risk infected person in 2000, including a small rate of infectious importations in the future, but no active screening. For each scenario, the top panel shows the distribution of the total number of infected people in time and the lower panels show the distribution between the risk groups at selected time points. The red line in the top panel shows the mean expected dynamics and the red crosses in the bottom panels show the expected numbers in each risk class at that time.

Solving the Kolmogorov forward equations, using the matrix exponential in the form presented in equation (2.7), we can obtain projections of how the infection dynamics will change in time in full probabilistic form. As an illustrative example, we calculate the distribution of infection up to the year 2030 for a village of 1000 people in a high-incidence health zone, assuming that within the village, there is an active screening coverage of 50% every year (figure 2*a*). We are assuming a small rate of infectious importations from movement of people that decreases with time, *δ*(*Y*) = (3.4 × 10^−6^) exp(− 0.1071(*Y* − 2000)) d^−1^, and that the village starts at endemic equilibrium conditions in 1998, calculated as the steady state of the Kolmogorov forward equations.

The results show that the introduction of active screening drives down the expected number of infected people from the steady-state distribution in 1998. By 2030, there is a probability of 0.90 that there are no infected people in this example village. The steady state is concentrated on negligible infection levels assuming the active screening is maintained. The full probability distribution does, however, show that for this individual village, there is still some probability the infections will not fall as quickly, or indeed remain constant or increase.

These results are dependent on the import rate between villages. Without the importation rate, there is an absorbing disease-free population state dynamics, which the population would eventually move towards (a steady state of no infection). This is because without the importation rate, if infection levels reach zero there is no person to re-introduce infection to the population (albeit through tsetse in practice).

Conversely to an endemic village, we consider a hypothetical village in a high-incidence health zone that was infection-free in 1998 and therefore not targeted for active screening (figure 2*b*). We predict some level of resurgence on average in the village if a single high-risk person of the village became infected through travelling to another village. There is a high probability of onward transmission in the village; the expected number of infected people increases initially, before decreasing again. However, infection is unlikely to be maintained in the long term, even without additional controls—there is a probability of 0.83 of a return to no infection by 2030.

### 3.2. Infection dynamics across multiple villages

The infection dynamics in individual villages informs us about the probability of local elimination of infection for an average village, but at a larger scale, such as health zone or country level, elimination of infection will depend on more than the probability of elimination in individual villages. While the distribution of gHAT infection is heterogeneous across the DRC [29], highly clustered incidence means that local movement of people will affect the probability of elimination in neighbouring villages. The rate of infectious importations in a village will be dependent on the total infection level in the area.

Therefore, to consider the dynamics across multiple villages in a region, we modify our rate of infectious importations to remove the exponential decrease in time matched to the trend in global infections and replace this with a term proportional to the total number of expected infections in model predictions across all villages of the local study region such that *δ*(1998) = (3.4 × 10^−6^) d^−1^. The electronic supplementary material provides a complete description of how the rate of infectious importations is formulated.

**Figure 3.**
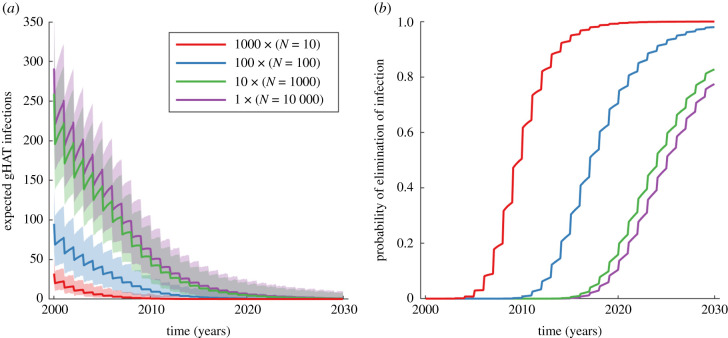
Infection dynamics in a total population of 10 000, partitioned into groups of villages of different sizes *N* = 10 000, 1000, 100 and 10. The annual active screening coverage is 50% and projections are started for the equilibrium distribution in 1998. (*a*) The expected number of infections. The shaded region shows the 95% prediction intervals around the solid line for the expected value. (*b*) The probability of local elimination of infection.

We consider the expected number of infections and the probability of elimination of infection for four groups of 10 000 people, comprised of groups of villages of different sizes (*N* = 10 000, 1000, 100 and 10) (figure 3) to understand the impact of the metapopulation structure [23]. The smaller village population sizes within total populated area have fewer expected gHAT infections and a higher probability of elimination of infection. The reduced number of interactions of mixing in smaller villages also results in a lower steady-state from before the active screening begins. There is a much lower probability of elimination of infection when there are fewer larger villages. For 1000 villages with population size *N* = 10, the probability of elimination of infection in 2030 is >0.99, while for just one village of size *N* = 10 000, there is a smaller probability for elimination of infection by 2030 at 0.77. This is in agreement with results of metapopulation studies, where more stochastic fade-outs of infection occur in the smaller populations, leading to a greater probability of elimination of infection across larger areas when sub-divided into more populations [30]. The example here highlights the benefit of modelling the full stochastic dynamics, where the small population sizes determine the frequency of extinction events; the results from an ODE model with constant population size would not vary with the size of the population.

### 3.3. Infection dynamics across a health zone

While we have considered theoretical villages to explore the behaviour of the Kolmogorov forward equation model, we now use data from the WHO HAT Atlas [31] to obtain a list of real village size distributions to apply our model to. Since our model for a high-incidence health zone uses parameters matched to the data from the health zone of Kwamouth, we extract the population sizes of each village in Kwamouth (see electronic supplementary material for details) along with the past active screening coverage. A plausible screening pattern is obtained taking the mean active screening coverage across all screenings and the probability that any particular village listed in the WHO HAT Atlas is screened in a given year. For Kwamouth, these values are a 68.6% coverage occurring at a probability of 0.23 each year using active screening data from 2000 to 2018 (see electronic supplementary material for details). This active screening scheme is incorporated into the model by a new parameter for the probability of an active screening event in a village. Thus, we model active screening as the linear combination of the probability of no active screening multiplied by the current distribution and the probability of active screening multiplied by the distribution after an active screening event. We note that this is not expected to reproduce the transmission and reporting trends of the past 19 years robustly—as we do not use screening patterns specific to individual villages, nor allow for coverages higher or lower than the mean—however, this does allow us to investigate general behaviour of the infection system using plausible real-world-like screening patterns.

We additionally adapt the value of the rate of importations of infection at steady state (previously taken from Davis *et al.* [19]). We calculate the value that is now specific to the health zone of Kwamouth as *δ*(*Y*) = 2.86 × 10^−6^ d^−1^, which is determined by matching the steady state of the whole health zone to the steady state of the system of ODEs in the original model for Kwamouth.

**Figure 4.**
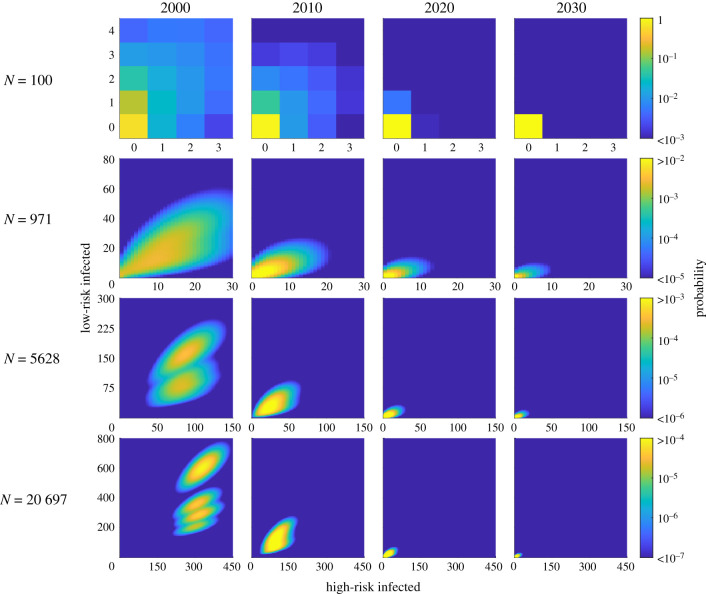
The risk distribution of infection in selected villages of an exemplar high-incidence health zone at different time points (the years 2000, 2010, 2020 and 2030); the first possible active screening of each village was in 1998. There are 418 villages with populations ranging between 3 and 20 697 of which we present the probability distribution of infected people in four villages (*N* = 100, 971, 5628 and 20 697). On each subplot, the *x*-axis represents the number of high-risk people infected, and the *y*-axis, the number of low-risk people infected. The risk distribution of infection for the alternative, low-incidence health zone is presented in the electronic supplementary material.

**Figure 5.**
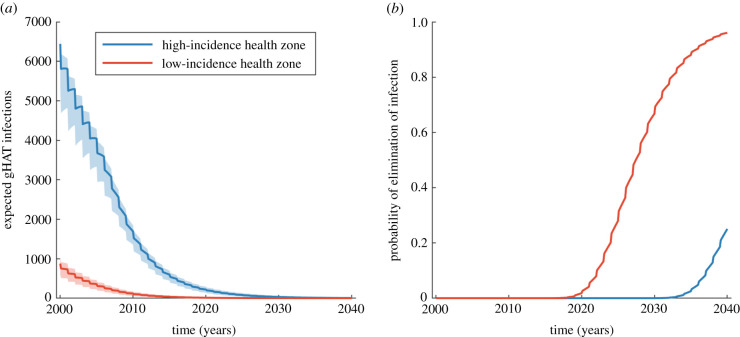
The total infection in the exemplar high-incidence and low-incidence health zones. We assumed the high-incidence health zone had a population of 206 135 people made up 481 distinct villages, while the low-incidence health zone had a population size of 107 685 of 204 villages. (*a*) The expected number of infections across all villages in time. The shaded region shows the 95% prediction intervals. (*b*) The probability of zero infections in time and hence elimination for all villages in each health zone together.

Applying the full Kolmogorov forward equation model to the high-incidence health zone, we obtain a full probability distribution in time for each village. We present the probability distribution of infection across the risk groups for a selection of villages of different sizes (*N* = 100, 971, 5628 and 20 697) at key time points (figure 4) and for the whole health zone across all simulated time (figure 5). Similar results for the lower-incidence health zone are presented in the electronic supplementary material.

The expected number of infections in all villages decreases in time, such that by 2030 most of the probability is centred around no infection. For the smallest villages, there is a large probability of local gHAT elimination by 2030 (greater than 0.99 for the village of size *N* < 100) as there are initially few or no cases, which are then identified by active screening, or passive screening and treatment or death. However, for the largest villages, such as the one with a population of *N* = 20 697, there is a high probability of continued infection with a probability of just 0.26 that elimination of infection will be met in that village by 2030. Hence, we will frequently see local elimination events, with global persistence across the health zone [30]. Additionally, we note that our results are not unique to this approach and the dependence on population size for the probability of elimination can be approximated in an ODE framework by imposing different thresholds for elimination [21].

Active screening is shown to reduce the infection. This is visually explicit in the column for 2000, where there are separate high probability clouds for whether an active screening has occurred and hence the number of infected people in the low-risk group identified and treated (figure 4). At the beginning of 2000, active screenings may have occurred in both 1998 and 1999 and so in the bottom left panel of figure 4 (village of population size *N* = 20 697), is is clear that there are four possible outcomes highlighted as separate regions of the probability distribution: an active screening event occurred in both 1998 and 1999, just 1998, just 1999 or neither 1998 nor 1999. This behaviour is less obvious in some smaller villages as the high probability regions overlap, yet is still present. We note that since active screening is identifying only low-risk individuals, infection is being pushed down but proportionally most of the reduction is in the low-risk group.

The decline in expected infection in all of individual villages is also evident in total infection of the health zone (figure 5). This is calculated as the sum of the expected infection in each village. Note that here we do not present the number of cases, but the underlying infections—case numbers would be substantially lower as there are typically high levels of under-reporting [32]. While our focus here is elimination, we additionally show how these levels of underlying infections correspond to annual active and passive case reporting for both high- and low-incidence exemplar health zones in the electronic supplementary material.

By 2030, the expected number of infected people have greatly decreased, yet persist in low numbers. This is mirrored in the probability of elimination of infection, calculated as the product of achieving zero infections in all villages of the health zone, which is less than 10^−4^ by 2030 in our high-incidence health zone if this active screening coverage remains constant and only identifies low-risk infected individuals, with the mean expected year of elimination after 2040. Using parameter values in the model matched to WHO HAT Atlas data for the low-incidence health zone, we observe an earlier mean expected year of elimination of infection in 2029.

## 4. Discussion

The Kolmogorov forward equation model facilitates a powerful and efficient way to analyse the dynamics of low-prevalence infections such as gHAT. This method presented here, using a lower-dimensional model structure than commonly used models, is fast to compute (with sufficiently small populations) and yet maintains a good correspondence with more complex approaches. The nature of the implementation means that various interesting properties can easily be explored, with exact methods for calculating extinction times and expected dynamics [6]. This approach has also allowed the model to be easily extended to consider the interaction of multiple villages and even to consider the dynamics of persistence at the health zone level by linking the total number of infected individuals to the rate of infectious imports into the villages.

Using this model, we conclude that based upon the strategy of active screening at the mean level, the expected year of elimination of infection may be beyond 2040 for high-incidence health zones like Kwamouth and around the target year of 2030 in low-incidence health zones like Mosango. As outlined in the methods, there are a few reasons why the exact results presented here may not completely align with the infection dynamics of specific health zones; this includes using mean screening coverage in villages, rather than village- and year-specific coverage, and the Kolmogorov forward equation model parameters have not been directly fitted to data, which would be needed to ensure a robust correspondence between observed outputs and reporting trends.

Improving the match to specific health zones is beyond the scope of the present study; however we do see that our general messages are in line with deterministic predictions using this parameterization but at a health zone level; Huang *et al.* [18] predicted that the year of elimination of infection would be after 2040 for Kwamouth and in 2031 for Mosango, using a similar model and the mean coverage of active screening. Likewise, stochastic models at a health zone level found that without COVID-19 interruptions to gHAT activities, elimination might be expected in 2025 in Mosango (with this slightly earlier prediction may be explained by higher assumed screening from 2017 onwards) [21]. This general agreement between either connected village scale and health zone scale models is reassuring (detailed further in the electronic supplementary material) and supports continued use of both model frameworks if acknowledging the stochastic and parameter uncertainty; however, by formulating connected village-scale methods it is possible to consider spatial dynamics in a more nuanced way.

We note that in considering the populations, we have only used villages that are listed in the WHO HAT Atlas and there are known to be additional villages within these health zones that are not listed, since they may not have ever been screened. Hence, the distribution of active screening is not truly as uniformly distributed across the health zones as presented here. As shown in the electronic supplementary material, we also know that screening coverage and frequency are correlated with population size of a settlement, and so adapting our model to account for this would also likely result in more accurate predictions. The proportion of people in each risk class is also constant for all population sizes in the model, whereas we could speculate that the larger populations are more town-like and perhaps have fewer high-risk members. This potential over-estimation of high-risk people in the large populations could explain some of the very high gHAT persistence probabilities seen in results for these populations (figure 4).

The assumption that movement of infected individuals is proportional to the expected number of people infected in the health zone, rather than the full distribution, is also a simplification to avoid calculating all possible combinations of infection states in each village. This simplification would be expected to reduce the size of the prediction intervals for small groups of villages, tending towards the mean behaviour; there would otherwise be a small probability of many villages having a much lower or higher number of infected people, which would decrease or increase the importation rate respectively. However, at the health zone level, with a large number of villages, the mean behaviour is a good approximation and so this simplification has minimal impact on the results.

In addition, we consider a probability of active screening every year, despite the fact that continued active screening is unlikely to be necessary for small villages, where the infection is almost certain to be locally eliminated in latter years. To improve the plausibility of the model, we could add a cessation criterion, similar other studies [20,33,34]. This is less straightforward to implement in this probabilistic framework than in the tau-leaping scheme, as we do not consider specific realizations of the model where the infection is either detected or not, but have a full probability distribution of all possible infection states. One potential solution could be to link the probability of being screened to the probability of observing a case in active screening. We do show that the assumption of a single screening event each year, as opposed to continuously throughout the year, shows negligible differences and so adopt this method for simplicity (see electronic supplementary material).

We have no data on the movement of people between villages, and so the rate of importation was estimated by matching to the probability of detecting infection on the first active screening in a village [19] or matching for the health zone to the expected equilibrium state of an ODE model variant [18]. However, using these values as an approximation for the mixing between villages, we achieve a good match to both ODE and event-driven stochastic (tau-leap) variants (see electronic supplementary material), while retaining the efficiency of the Kolmogorov forward equations and the additional benefits of calculating the full probability distribution. Explicit data on movement, such as a network structure for the amount of travel between villages, including travel outside the health zone, could refine our predictions, as well as capturing more of the heterogeneities between villages. However, low frequency of infectious imports to each village, coupled with minimal differences in the transmission dynamics between villages of the same health zone [19], mean we would not expect this to greatly influence predictions.

The deterministic ODE method is useful for large populations where we expect average behaviour, and the event-driven stochastic method is particularly useful for smaller populations, increasing our understanding of the stochastic uncertainty and ability to directly measure elimination. However, the Kolmogorov forward equation approach with connections between villages can be used to capture the dynamics of individual villages, with bespoke control interventions, with the results translatable between the village level and the health zone level, providing estimates of elimination of infection at practical spatial scales.

In the future, this modelling approach could be very valuable for assessing not only decisions about continuation or cessation of screening in specific villages (based on village size and previous detections) but also providing a method through which the impact of village-level mass drug administration on health zone transmission dynamics could be assessed. While such as drug is not currently licensed for this type of delivery, a new compound—acoziborole—which is in phase 3 clinical trials as a single-dose cure, is a possible candidate [35]. These types of village-scale strategy decisions would be challenging to analyse through health zone level approaches. In addition, while the tsetse are not explicitly modelled in our approach, our model is sufficiently flexible that future vector control in these health zones could be incorporated by a reduction in the force of infection due to a reduction in tsetse populations.

## 5. Conclusion

We have shown that a lower-dimensional model of gHAT that operates at the village level can achieve very similar results to a more biologically realistic version for larger spatial scales, while introducing a method of obtaining numerically exact results for extinction times and expected number of infected individuals. The predictions provided are in line with previous deterministic and stochastic results and the model implementation provides a framework to scale between modelling at a health zone or national level and at the village level. This is an important development, since the data obtained and the actual interventions (active screening) conducted are at village level.

The Kolmogorov forward equation model suggests that with mean coverage of active screening and continuing passive screening, with no additional interventions such as vector control, the infection is almost certain to persist for long periods. This indicates that additional or intensified controls are required to achieve elimination of transmission, such as tsetse control, improved passive detection or targeting of high-risk people in active screening. This model structure expands the range of analytical projections that it is possible to generate and demonstrates the results that can be obtained with a Kolmogorov forward equation model.

## Data accessibility

This work does not include any original data. Epidemiological data for the study were provided by the WHO in the frame of the Atlas of HAT which may be viewed at www.who.int/trypanosomiasis_african/country/risk_AFRO/en and may be requested through Jose Ramon Franco (francoj@who.int).
